# MS/MS-Based Molecular Networking: An Efficient Approach for Natural Products Dereplication

**DOI:** 10.3390/molecules28010157

**Published:** 2022-12-24

**Authors:** Guo-Fei Qin, Xiao Zhang, Feng Zhu, Zong-Qing Huo, Qing-Qiang Yao, Qun Feng, Zhong Liu, Gui-Min Zhang, Jing-Chun Yao, Hong-Bao Liang

**Affiliations:** 1 State Key Laboratory of Generic Manufacture Technology of Chinese Traditional Medicine, Lunan Pharmaceutical Group Co., Ltd., Linyi 273400, China; 2 College of Pharmacy, Shandong University of Traditional Chinese Medicine, Jinan 250355, China; 3 Jining Medical University, Jining 272067, China

**Keywords:** MS/MS-based molecular networking, natural products dereplication, classical MN (CLMN), feature-based molecular networking (FBMN), ion identity molecular networking (IIMN), building blocks-based molecular network (BBMN), substructure-based MN (MS2LDA), bioactivity-based molecular networking (BMN)

## Abstract

Natural products (NPs) have historically played a primary role in the discovery of small-molecule drugs. However, due to the advent of other methodologies and the drawbacks of NPs, the pharmaceutical industry has largely declined in interest regarding the screening of new drugs from NPs since 2000. There are many technical bottlenecks to quickly obtaining new bioactive NPs on a large scale, which has made NP-based drug discovery very time-consuming, and the first thorny problem faced by researchers is how to dereplicate NPs from crude extracts. Remarkably, with the rapid development of omics, analytical instrumentation, and artificial intelligence technology, in 2012, an efficient approach, known as tandem mass spectrometry (MS/MS)-based molecular networking (MN) analysis, was developed to avoid the rediscovery of known compounds from the complex natural mixtures. Then, in the past decade, based on the classical MN (CLMN), feature-based MN (FBMN), ion identity MN (IIMN), building blocks-based molecular network (BBMN), substructure-based MN (MS2LDA), and bioactivity-based MN (BMN) methods have been presented. In this paper, we review the basic principles, general workflow, and application examples of the methods mentioned above, to further the research and applications of these methods.

## 1. Introduction

As the result of millions of years of evolutionary optimization, natural products (NPs) have been endowed with privileged pharmacological functions, and historically became the most important source for drug discovery [1,2,3]. Among the 1394 small-molecule drugs approved by the United States Food and Drugs Administration (FDA) from 1981 to 2019, 31.6% were botanical drugs, unaltered NPs, and NP derivatives, and 30.4% were synthetic drugs with NP pharmacophores or the mimicry of NPs [4], which means that close to 2/3 of the small-molecule medicines of this period were associated with NPs. Meanwhile, in the top 200 pharmaceuticals by retail sales in 2021, NP-derived medicines were successful in the areas of antibiotics and antifungal, anticancer, cholesterol-lowering, immunosuppression, and antihypertensive properties [5,6]. Despite such tremendous success, it is noticeable that many pharmaceutical companies have terminated their programs to screen new chemical entities from NPs since 2000 [7,8,9]. The reasons given were the rapid advances of biopharmaceuticals [10], kinase-based drugs [11], antibody-drug conjugates (ADC) [12], proteolysis-targeting chimeras (PROTAC) [13], and other methodologies [14,15]. However, no fundamental breakthroughs to overcome the drawbacks of NPs have been made for some time [5,7,8,9], especially in terms of rapidly screening new and bioactive NPs from complex extracts; economically obtaining sufficient quantities of pure target compounds was the less widely advertised reason [8,9,16].

Indeed, with the large and increasing number of NPs (estimated at 600,000), rediscovery was commonplace in natural product research [16,17,18,19], and consequently, the problem of how to rapidly identify new NPs from complex mixtures has become a thorny challenge that needs to be resolved [18,19]. To circumvent this issue, a number of early prioritization strategies were achieved by manually comparing characteristics such as the ultraviolet-visible spectra (UV/Vis spectra), nuclear magnetic resonance (NMR), or mass spectra (MS) with various databases [20,21,22], or by tracking biological activity and other methods [23,24,25]. In practice, these methods were also accompanied by laborious, time-consuming procedures and high rediscovery rates [16]. With the recent rapid advances in analytical instrumentation and artificial intelligence technology, proteomics [26,27,28], genomics [29], metabolomics [30], and transcriptomics [31,32] have enabled tremendous achievements that greatly promoted and influenced the development of life sciences. In the past decade, the research method and technology of metabolomics and proteomics were also borrowed to prioritize the targeted isolates of NPs [33,34,35]. Since a major bottleneck in the omics pipeline is the annotation and identification of the spectral data, many spectral interpretation methods, such as MS- and/or NMR-based approaches, were developed [36,37,38,39]. Among them, tandem mass spectrometry (MS/MS)-based molecular networking (MN) has become an increasingly popular and attractive NPs research tool that integrated the advantages of sensitiveness, high throughput, and the robustness of MS/MS with the ability of MN to organize and visualize large MS/MS datasets [37,38].

The classical MS/MS-based MN (CLMN) was first reported by Dorrestein’s group in 2012 to investigate the metabolic profiles of living microbial colonies [40]. In 2013, the Global Natural Product Social Molecular Networking (GNPS, http://gnps.ucsd.edu) group, a sharing and community-based web platform used to store, analyze, share, and compare MS/MS data, as well as perform the generation of MN [41,42], further promoted the development of MN in different research groups. Currently, this technique is widely used, such as in the study of forensics [43], food chemistry [44], environmental science [45], plant science [46], and others [47,48]. In natural product research, on the basis of CLMN, the feature-based MN (FBMN) [42], ion-identifying MN (IIMN) [49], building blocks-based molecular network (BBMN) [50], substructure-based MN (MS2LDA) [51], and bioactive MN (BMN) [52] were presented in the form of different interpreting methods of the obtained data. In this paper, we review the basic principles, general workflow, and application examples of the above-mentioned methods, aiming to promote further research and applications.

## 2. Classical Molecular Networking (CLMN)

**Figure 1:**
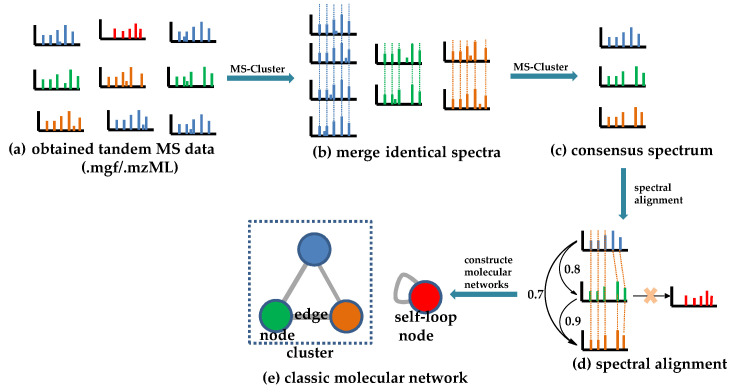
Schematic representation of the principle of CLMN. (**a**) The obtained tandem MS data. (**b**) The merging of identical spectra. (**c**) The consensus spectrum. (**d**) Spectral alignment. (**e**) The classic molecular network.

The theoretical rationale of CLMN is that molecules with similar structures will exhibit considerable similarities in their MS/MS spectra, and vice versa. Thus, similar molecules in complex mixtures can be clustered to form “molecular families” by the mass spectral similarities of molecules. The spectral similarities can be calculated with a vector-based modified “cosine score” (ranging from 0 to 1; the higher the score is, the more similar the result will be), which takes into account the number of matching fragment ions, the relative intensities of the peaks, and the parent mass accuracy [53]. As shown in Figure 1, the obtained tandem MS spectra (Figure 1a) are first processed to give a consensus spectrum (Figure 1c) by merging identical spectra (Figure 1b), using the MS-Cluster algorithm to avoid identical spectra appearing more than once [25,53]. Then, a modified algorithm is used to calculate the spectral similarity score (Figure 1d). Peaks from one consensus spectrum are compared with peaks from the other, either at identical *m/z* values or with their Δ*m/z*, considering that a Δ*m/z* change to the precursor ion may lead to shifting a subset of fragment peaks by Δ*m/z* [36,53]. Finally, a molecular network is constructed on the basis of the calculated spectral similarity score (Figure 1e). In the network, the “molecular families” and the “molecular only similar with itself” variables are represented by “cluster” and “self-loop node”, respectively. In the cluster, similar molecules (“node”) are connected by lines (”edge”), and the thickness of the edges showcases the level of their similarity [53].

**Figure 2:**
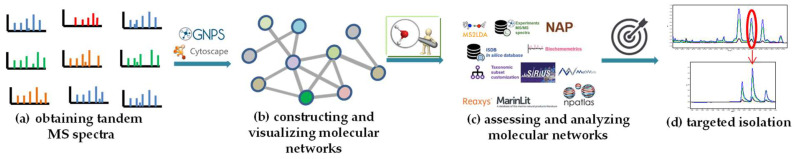
Schematic workflow for CLMN in NP dereplication. (**a**) Obtaining the tandem MS spectra. (**b**) Constructing and visualizing molecular networks. (**c**) Assessing and analyzing molecular networks. (**d**) Targeted isolation.

A schematic workflow for a CLMN dereplication pipeline is presented in Figure 2. There are four main steps: (Figure 2a) obtaining the tandem MS spectra; (Figure 2b) constructing and visualizing the molecular networks; (Figure 2c) assessing and analyzing the molecular networks; (Figure 2d) targeted isolation (Figure 2) [25]. As the tandem mass spectrometry experiments for data acquisition represent one of the most important factors affecting molecular networks, all samples should be prepared and analyzed in the same way [24]. After uploading the obtained tandem MS spectra to the GNPS platform, the completed job can be visualized either in the platform [41] or in Cytoscape [54]. A detailed protocol from the tandem mass spectrometry experiments, via a publishable and reproducible molecular network in the GNPS platform, has been provided by Dorrestein et al. in *Nature Protocols* [53], and we can refer to this protocol here. Another tool to generate and visualize molecular networks is the MetGem software (https://metgem.github.io, accessed on 7 November 2022) [55], which was developed based on the t-distributed stochastic neighbor embedding (t-SNE) algorithm in 2018, a well-known visualization technique used for high-dimensional data [56]. The t-SNE-based MetGem allows clustering spectra, relying on local details within the entire data space rather than individual links between spectra, and can thereby avoid having too many “self-loop nodes” or fusing “molecular families” in the molecular networking when a similarity cutoff is set [36]. However, the t-SNE-based method could not offer information about the relationships between “nodes”, and it is complementary to the cosine similarity-based classic GNPS-style MN [36]. More recently, deep learning mass spectral similarity scoring methods have also been developed, such as Spec2Vec and MS2DeepScore, which derive abstract spectral embeddings to assess spectral similarity by learning the fragment relationships among large amounts of spectral data [57,58]. Of course, mismatches between the calculated mass spectral similarity scores and the true structural similarities are very common, and the comprehensive use of multiple methods can reduce those mismatches.

**Figure 3:**
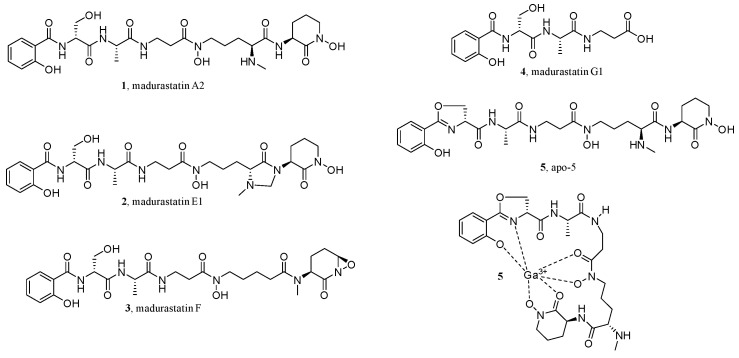
Structures of new madurastatin derivatives.

As CLMN can visualize and map the chemical space in the extracts of organisms, it is widely used to determine the preferred species [59,60], the culture conditions of microorganisms [60], isolation workflow [61], etc. For example, in searching for siderophores from *Actinomadura* sp. RB99, activity assays and MS/MS-based CLMN were used as the dereplication strategies [59]. First, after co-culturing with *Pseudoxylaria* sp. X802, the extracts obtained from the colony of RB99 and the interaction zone of inhibition, as well as RB99 cultures grown on different media, were analyzed by an high-resolution electrospray ionization tandem mass spectrometry (ESI-HRMS^2^)-based MN. The obtained GNPS network suggested chemical diversity and dereplicated clusters of phosphoethanolamines, phosphocholines, oligosaccharides, pseudoxylallemycins, and cytochalasins, together with an interesting small GNPS cluster. Further analysis of the proposed molecular formulas of the interesting small cluster indicated structural changes of -O and -CH_2_ and a peptidic backbone, with a putative *N,O*-ratio characteristic for siderophores. Then, based on these findings and the optimized cultivation conditions, the up-scaled refermentation of RB99 led to the isolation of five new madurastatin derivatives (**1**–**5**), including a siderophore-metal complex (**5**) (Figure 3).

## 3. Feature-Based Molecular Networking (FBMN)

**Figure 4:**
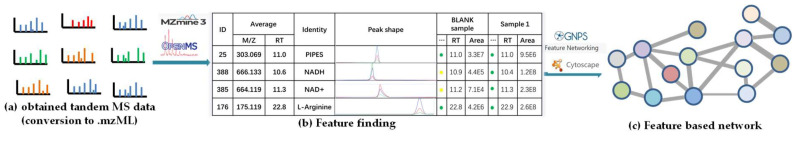
Schematic representation of the principles of FBMN. (**a**) The obtained tandem MS data. (**b**) Feature-finding. (**c**) The feature-based network.

Although CLMN is very convenient for the rapid processing of large-scale MS/MS datasets, it cannot differentiate positional isomers or stereoisomers, or provide accurate relative quantitative information, due to the limitations of the MS-Cluster algorithm. To address this issue, Dorrestein’s group developed FBMN by integrating comparative metabolomics with MN in 2017 [42]. In this method, not only the fragmentation data but also the isotope patterns, the retention times, and the ion mobility spectrometry can be compared. Compared with CLMN, there are two main different steps in the workflow of FBMN (Figure 4). First, the obtained tandem MS spectra (Figure 4a) should be pre-processed using MZmine [62], OpenMS [63], or other feature detection and alignment tools [42] to detect, group, and align those features (Figure 4b). Second, the exported feature lists ((.cvs, feature quantification table) and (.mgf, MS^2^ spectral summary file)) are uploaded to perform the dedicated feature networking workflow on the GNPS platform, to generate a feature-based network (Figure 4c) [42].

**Figure 5:**
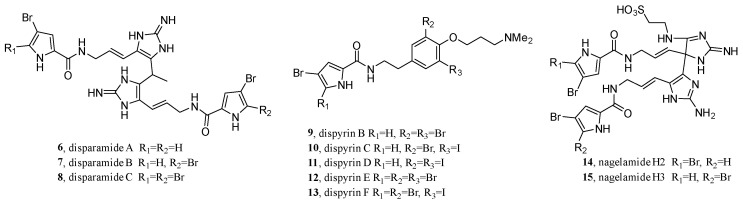
Structures of disparamides A–C and seven other new compounds.

Limited by the chromatographic feature-finding tools and different experimental conditions, FBMN is especially suitable for one or a few samples and has become the second most commonly used tool in GNPS [42]. In revisiting the bromopyrrole alkaloids of the extensively investigated marine sponge, *Agelas dispar*, FBMN was used by Berlinck’s team as the dereplication strategy [64]. After separation by extraction and C_8_ RP column chromatography, the defatted EtOH/MeOH extract of *A. dispar* was divided into five fractions. Then, three fractions with brominated compounds were subjected to Sephadex LH-20 to yield 63 fractions, which were further analyzed by quadrupole time of flight (QToF)-MS/MS to generate a feature-based molecular network. Finally, after dereplication with the in-house and in silico database (ISDB), clusters of undescribed compounds were selected for study; this resulted in the isolation of disparamides A–C (**6**–**8**, with a novel carbon skeleton) and seven other new compounds (**9**–**15**) (Figure 5).

## 4. Ion Identity Molecular Networking (IIMN)

**Figure 6:**
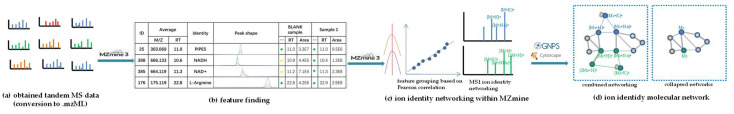
Schematic representation of the principle of IIMN. (**a**) The obtained tandem MS data. (**b**) Feature-finding. (**c**) Ion identity networking within MZmine. (**d**) The ion identity molecular network.

In 2021, Dorrestein’s group further employed IIMN to overcome the disconnected sub-networks of “molecular families”, which was caused by limitations inherent to the different fragmentation behavior of the multiple ion species of a given compound [49]. For example, the two ion species of the same molecule, [M + H]^+^ and [M + Na]^+^, typically stay unconnected in a molecular network. As all ions from the same molecule can be connected, based on the known mass differences of ion species, an additional MS^1^ ion identity networking layer was fused to FBMN to create IIMN networks (Figure 6). Compared with FBMN, in the progress of detecting and aligning features with MZmine, one more feature list (a .csv file of ion identity networking (Figure 6c)) should also be exported. Although those three feature files are also uploaded to the FBMN, the resulting data of the IIMN are different from those of the FBMN; two .graphml networking files containing three networks (FBMN networks, IIMN networks, and collapsed IIMN networks (Figure 6d)) will be generated. Recently, this method, combined with activity profiling, was used for the prioritization of compounds inhibiting AK strain-transforming kinase (AKT) in human melanoma cells [65].

## 5. Building Blocks-Based Molecular Network (BBMN)

**Figure 7:**
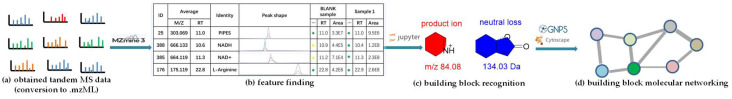
Schematic representation of the principle of BBMN. (**a**) The obtained tandem MS data. (**b**) Feature-finding. (**c**) Building-block recognition. (**d**) Building-block molecular networking.

**Figure 8:**
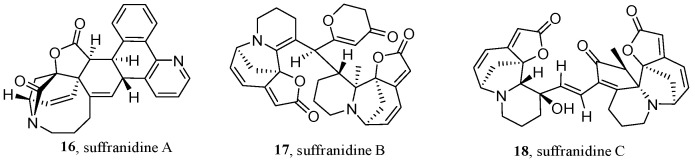
Structures of the novel *securinega* alkaloids.

Natural products are usually formed by biosynthetic pathways from specific building blocks. In 2021, Ye’s group first presented a BBMN to facilitate the efficient discovery of novel *securinega* alkaloids from *Flueggea suffruticosa* [50]. This method combined the neutral loss/product ion-scanning strategy [66] and MN, which can selectively identify biogenetically relevant secondary metabolites and optimize the bulky MS^2^ data by further filtering the features files in the pre-processing of the MS dataset (Figure 7). In this research, the EtOH extract from the twigs and leaves of *F. suffruticosa* was acidified with 10% HCl, extracted using CH_2_Cl_2_, basified using NH_4_OH, and re-extracted with CH_2_Cl_2_ to yield a total alkaloid. The acquired and deconvoluted ultra high performance liquid chromatography (UHPLC)-MS^2^ data (7a) of the total alkaloid comprised features extracted with MZmine (Figure 7b). Then, *m/z* at 84.08 and 134.03 Da were selected as the product ion and the neutral loss, to detect the building blocks of *securinega* alkaloids from the obtained features (Figure 7c), and were then filtered using a Python script, respectively. Subsequently, the BBMNs were constructed by GNPS and MetGem (Figure 7d) and annotated by an in-house library. Then, three unknown and unclustered nodes with *m/z* values over 400 were selected as the target compounds. Finally, LC-MS-guided isolation led to the isolation of three novel *securinega* alkaloids (**16**–**18**) (Figure 8).

## 6. Substructure-Based Molecular Networking (MS2LDA)

**Figure 9:**
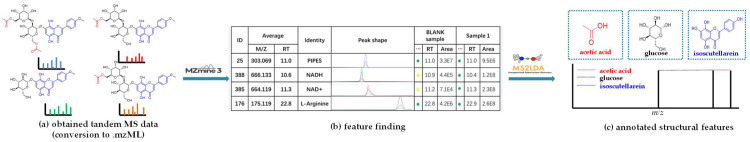
Schematic representation of the principles of MS2LDA. (**a**) The obtained tandem MS data. (**b**) Feature-finding. (**c**) The annotated structural features.

Apart from the efficient use of MS^2^ datasets, the spectral annotation of molecular networks is another algorithmic bottleneck. It has been noted that many strategies, such as the In Silico MS/MS DataBase (ISDB) [67], network annotation propagation (NAP) [68], Sirius [69], MetWork [70], and MS2LDA [51,71] have been proposed. In 2016, inspired by the text-mining algorithm for latent Dirichlet allocation (LDA) [72], Hooft and colleagues presented an MS2LDA that can find and match co-occurring molecular fragments and neutral spectra (“Mass2Motifs”) from fragmentation spectra (Figure 9a) in an unsupervised manner [36,51] (Figure 9). In turn, the obtained Mass2Motifs provide information on the functional groups, building blocks, or even scaffolds of a compound (Figure 9c) that can be used for the de novo annotation of unknown molecules without reference spectra. Other substructure discovery-based tools, including the metabolite substructure auto-recommender (MESSAR) [73], and CSI (compound structure identification):FingerID [74] were also developed. Among them, MESSAR is a complementary approach to MS2LDA that can provide an automated structural annotation of Mass2Motifs.

**Figure 10:**
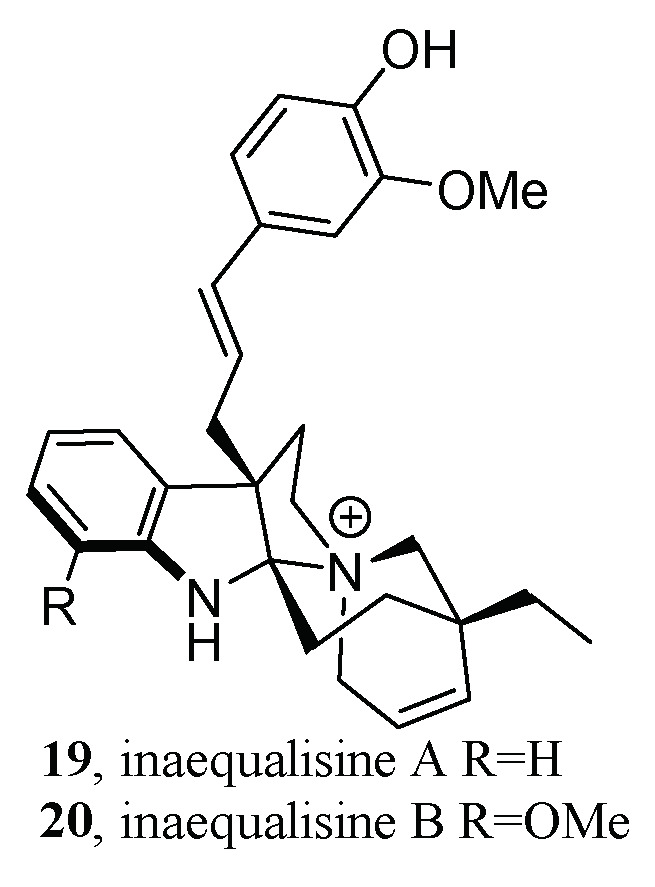
Structures of two novel hybrid alkylated phenylpropane MIAs.

For example, in the search for monoterpene indole alkaloids (MIAs) from the roots of *Callichilia inaequalis* Stapf (Apocynaceae), MS2LDA was employed to fine-tune the isolation workflow [75]. The obtained MS^2^ data of the seven alkaloid fractions were processed by FBMN. After MIADB [76] spectral library annotation, some clusters of the molecular network remained unannotated; thus, MS2LDA was utilized to further unearth more information from these clusters. As a result, molecular family A, a nine-parent mass shared cluster, exhibited an intriguing mass loss of 162.075 Da that did not match any MotifDB. A further literature survey indicated that this Mass2Motif may be a hexose unit, but this annotation was inconsistent with both the molecular formulas and the elemental composition. Hence, molecular family A was selected for targeted isolation, then liquid chromatography (LC)-diode array detector (DAD)-MS-evaporative light scattering detector (ELSD)-guided isolation led to the discovery of two novel hybrid alkylated phenylpropane MIAs (**19**–**20**) (Figure 10).

## 7. Bioactivity-Based Molecular Networking (BMN)

**Figure 11:**
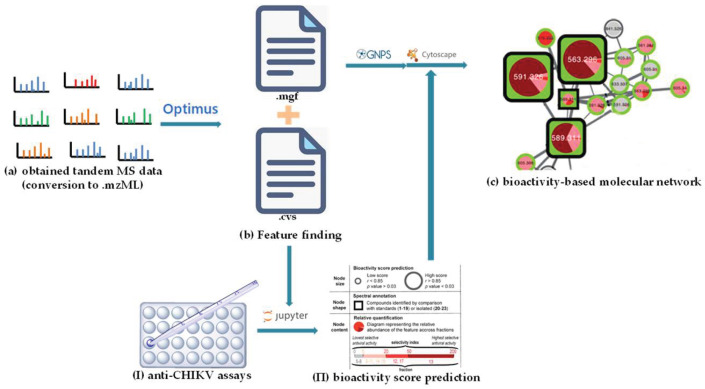
Schematic representation of the principle of BMN. (**a**) The obtained tandem MS data. (**b**) Feature-finding. (**I**) Anti-CHIKV assays. (**II**) Bioactivity score prediction. (**c**) The bioactivity-based molecular network.

**Figure 12:**
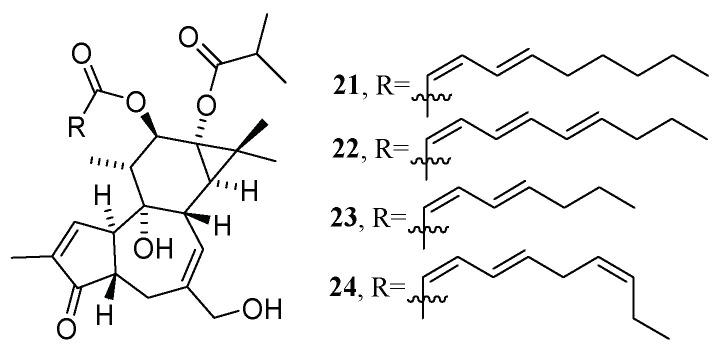
Structures of four new 4*β*-deoxyphorbol esters.

In addition, other NP prioritization strategies were also developed, based on the combination of other layers of information, such as biochemometrics [77], genomics [78], and taxonomy [79]. In the search for bioactive compounds against the chikungunya virus (CHIKV) from *Euphorbia dendroides*, Dorrestein’s group presented BMN by combining chemometrics with MN in 2018 [52]. Chemometrics can distinguish the active and inactive compounds in mixtures but cannot provide structural information regarding them [80]. In this study, after chromatographic separation, the obtained 18 fractions of the *E. dendroides* latex extract were detected to obtain their LC-MS/MS data (Figure 11a) and anti-CHIKV activities (Figure 11I). The MS^2^ data were pre-processed by Optimus [81] to generate the spectral features files (Figure 11b) (the .mgf file and the .cvs file, which were used to generate CLMN and bioactivity scores, respectively) (see Figure 11). The bioactivity scores for each ion were calculated using a Pearson correlation that correlated the relative abundance of an ion and the selectivity index of fractions (Figure 11**Ⅱ**). Finally, combined with the predicted bioactivity scores, the bioactive molecular network was generated using GNPS (Figure 11c), in which a large node size indicated high activity, and relative abundance in a certain fraction was shown by a pie chart. A detailed analysis of the molecular networks indicated that the compounds in molecular network 2 were most likely to be promising bioactive candidates. Then, four new 4*β*-deoxyphorbol esters (**21**–**24**) (Figure 12) with anti-CHIKV activities were obtained by HPLC-guided isolation.

## 8. Conclusions

Needless to say, NPs have played an important role in the discovery of small-molecule drugs in the past. According to Linington’s analysis [18], the future for NPs is very bright, and the chemical space is large. The launch of ADC [12] and the industrialization of Eribulin (Halaven^TM^) [82] showed us the infinite possibilities for new drugs from NPs. Nevertheless, aggressive innovation is needed to tackle bottlenecks such as the rapid discovery and large-scale availability of NPs. In the past decade, the advent of MS/MS-based MN has greatly promoted the development of NP dereplication methods. In this review, we introduced CLMN, FBMN, IIMN, BBMN, MS2LDA, and BMN, along with their basic principles, general workflow, and application examples, hoping to further the research and applications of these methods. Although there are numerous studies of MN, such as those on datasets, algorithms, data pre-processing, annotation, and the different types of mass spectrometers or hybrids with other methods, this review covers the key concepts and steps in the molecular network construction pipeline, which is helpful for beginners hoping to learn this methodology.

It is worth noting that MS-based analysis is a biased detection method, depending on how well the compounds fragment, and some NPs do not ionize as well as others. NMR-based approaches can make up for this shortcoming, and dedicated 2D-NMR-based prioritization strategies have been developed rapidly [83,84,85]. As the two techniques can complement each other, new hybrid MS/NMR approaches are emerging for NPs prioritization [39]. On the other hand, although many annotation methods have been developed, the exploitation of molecular networks still requires manual intervention and expertise. We believe that open access to data, including both the MS and NMR spectra, and advancements in big data approaches will improve the efficiency of NPs dereplication.
